# Can the *Normic de minimis Expected Utility Theory* save the *de minimis* Principle?

**DOI:** 10.1007/s10670-023-00751-x

**Published:** 2023-11-28

**Authors:** Björn Lundgren, H. Orri Stefánsson

**Affiliations:** 1 Department of Philosophy and Religious Studies, Utrecht University, Utrecht, The Netherlands; 2 Institute for Futures Studies, Stockholm, Sweden; 3 Department of Philosophy, Stockholm University, Stockholm, Sweden; 4 Swedish Collegium for Advanced Study, Uppsala, Sweden

## Abstract

Recently, Martin Smith defended a view he called the “normic de minimis expected utility theory”. The basic idea is to integrate a ‘normic’ version of the de minimis principle into an expected utility-based decision theoretical framework. According to the de minimis principle some risks are so small (falling below a threshold) that they can be ignored. While this threshold standardly is defined in terms of some probability, the normic conception of de minimis defines this threshold in terms of abnormality. In this article, we present three independent arguments against the normic de minimis expected utility theory, focusing on its reliance on the de minimis principle.

## Introduction

Recently, Smith (2022) defended a view he called “normic de minimis expected utility theory” (NDEUT). The basic idea is to integrate a ‘normic’ version of the *de minimis* principle into an expected utility-based decision theoretical framework. According to the *de minimis* principle some risks are so small (falling below a threshold) that they can be ignored (Smith, 2022; Lundgren & Stefánsson, 2020; Adler, 2007; Peterson, 2002).

Standardly, the *de minimis* threshold is defined in terms of some probability.^1^ However, Smith uses the concept of normic risk, according to which risk—and in this case, the *de minimis* threshold—is defined in terms of abnormality (Smith, 2022; see also Ebert et al., 2020). Simply put, the more abnormal it would be for an action to result in some undesirable outcome, the less risky it is that the action results in that outcome.

In this paper, we challenge NDEUT. We give three independent arguments, each of which we think call in question the normative validity of NDEUT. Our focus here is on the normic conception of the *de minimis* principle, which is an essential part of the framework. First, we start by building on a previous argument against the standard—probability-based—*de minimis* principle advanced by Lundgren and Stefánsson (2020). We show that their argument can be applied to NDEUT as well. Specifically, we show that NDEUT violates statewise dominance (for seemingly no good reason). Second, we turn to argue that NDEUT implies that we are permitted to ignore risks that have a cost-efficient solution (for similar problems regarding a probability-based notion of *de minimis*, see Lundgren & Stefánsson, 2020; Mumpower, 1986). Third and lastly, we argue that in some cases abnormality and disutility will come apart such that the intuitively most devastating risks are treated as the most abnormal and thus *de minimis* (i.e., it is permissible to ignore them).

The paper is structured as follows. We start by presenting NDEUT. Next, we present some of Lundgren and Stefánsson’s arguments against the probability-based *de minimis* principle. After all of the necessary parts are in place, we present our counterexamples to NDEUT. We end the article by summarizing our arguments.

## The Normic de minimis Decision Theory

Although Smith’s paper contains a rich and complex discussion, our presentation will be limited to the *de minimis* principle’s role in NDEUT, which depends on the concept of normic risk.

Normic risk differs from standard conceptions of risk, according to which a risk is evaluated in terms of the probability of the risked outcome or event (typically in conjunction with the outcome or event’s severity, that is, negative utility). The concept of normic risk is not defined in terms of probability but in terms of *normalcy*. Although the term ‘normal’ is sometimes used colloquially to imply that something is probable or frequent, Smith gives the following example to illustrate the difference:

Suppose you’re trying to decide whether to take the bus home and I remark ‘the bus ride wouldn’t normally take more than 20 minutes’. Part of what I’m saying here is that circumstances would have to *conspire against you* in some way in order for the ride to take more than 20 minutes—it would have to be that the bus breaks down, or runs out of petrol, or gets stuck in traffic, or is diverted by roadworks etc. but, absent any of these interfering factors, the trip would take 20 minutes or shorter. Put differently, if you get on the bus, and the trip ends up taking longer than 20 minutes, there would have to be some *special explanation* as to how this happened. In contrast, if your opponent [in a poker game] happened to be dealt a hand that beats your three-of-a-kind then, while you may think yourself unlucky, no special explanation is needed for this. When we say that a given outcome would be abnormal, what we are sometimes claiming is that there would have to be some special explanation if it were to result from the action in question […]. It is this notion of abnormality that is appealed to in the normic account of risk. (Smith, 2022; our modifications within brackets)^2^

Moreover, “the risk of a possible outcome is determined by its *abnormality*. More precisely, the risk that a particular outcome would result from an action, given the agent’s evidence, depends upon how abnormal it would be for the outcome to result from the action, given the agent’s evidence” (ibid).

Although the concept of normalcy is a bit vague, this explanation will suffice for the present purpose. Simply put, under NDEUT, a risk is *de minimis*—and can therefore be ignored—if it is sufficiently abnormal.

Smith motivates the usage of the normic understanding of *de minimis* by pointing out that it can help resolve some problems regarding the standard probabilistic conception. For example, suppose the *de minimis* threshold is 1/15,000 and that there is a 1/10,000 chance that there is asbestos in a house, which in turn depends on a 1/20,000 chance for each of two different types of asbestos. Should we include or exclude these risks? (Smith, 2022) Since probabilities are *additive*—that is, the probability that there is asbestos of the one type or the other type equals the sum of the probability that there is asbestos of the one type plus the probability that there is asbestos of the other type (assuming that the two types are mutually exclusive)—a probabilistic *de minimis* threshold will imply that we can ignore the risk when we consider each type of asbestos separately, but not if we consider them together. So, whether we should be free ignore the risk or not depends on how we formulate the decision problem.^3^ Normalcy is not additive, however: there being asbestos of one or the other type in the house is just as abnormal as the occurrence of the type of asbestos that would be *less* abnormal. So, the normic *de minimis* account will not imply that we either can ignore the risk or not depending on how we set up the decision problem. And that, as Smith points out, seems to count in favor of the normic *de minimis* account over the probabilistic one.^4^

However, while normic risk “determines which outcomes have to be included in the calculation”, Smith says that “probabilistic risk continues to determine how each of the outcomes in an expected utility calculation is weighted” (Smith, 2022). So, NDEUT is not a complete departure from orthodox expected utility theory.

## Statewise Dominance and the *De Minimis* Principle

Lundgren and Stefánsson (2020) present a few arguments against a probability-based conception of the *de minimis* principle. In this paper, we focus on the violation of statewise dominance. Consider a situation in which we have two options (A and B). Suppose that we know with certainty that A and B are identical options except that A has no risk and B has a *de minimis* risk. This can be illustrated using the following matrix (the numbers and states are from Lundgren & Stefánsson, 2020):

**Table:** 

	s1	s2	s3
A	1	1	1
B	1	1	-1

Suppose that the third state, s3, falls below the *de minimis* threshold, understood either probabilistically or in terms of normalcy. Then, according to proponents of the *de minimis* principle, A and B are equally good. However, A statewise dominates B, which suggests that they are not equally good. So, we contend, the *de minimis* principle cannot be part of a normatively valid decision procedure.^5^

To demonstrate that this problem can realistically arise, Lundgren and Stefánsson present an example—which we will build on below in our analysis of NDEUT—where a risk analyst is instructed to rank different types of paint, to be used for a playground, according to their health risks. As they note, given the limited criteria of evaluation it seems plausible that one type of paint could increase the risk of cancer by some *de minimis* probability and that the paints could otherwise be identical. Now, suppose that the paint causes cancer in children who have some extremely *abnormal* genetic condition. (The original argument referred to a *rare* condition, but abnormality works just as well.) Then s3 can be interpreted as a state where a child with this condition enters the playground in question. (p. 913) Thus, we have a realistic example where the *de minimis* principle—construed either in terms of probability or normality—violates statewise dominance.

Lastly, as Lundgren and Stefánsson point out, this example also shows that addressing *de minimis* risks can sometimes be cost-effective (cf. Mumpower, 1986). For instance, if two types of paints are just as expensive and equally widely available, and moreover equivalent in all respects except that one involves a *de minimis* risk, then eliminating the *de minimis* risk would arguably be free. Again, this argument works just as well against a *de minimis* principle construed in terms of abnormality as against a probabilistic *de minimis* principle.

In response to a different problem, Smith says that “NDEUT will always *allow* us to set a threshold in such a way that this outcome will not count as a de minimis risk” (Smith, 2022). This, however, will not help address the problem we have raised. For whatever the threshold should be in the given case, let the normalcy of the cancer risk be lower. Responding that NDEUT will allow us to set a threshold that will be lower than whatever is the case in the given situation means that such a threshold will not be very helpful in decision-making because then, in practice, the *de minimis* principle just says that you can exclude pretty much whatever you think you should exclude. Of course, there are still some restrictions, since one cannot exclude possibilities that are at least as normal as possibilities that are included or any maximally normal possibility. However, our point here is that the principle does not seem to help us to draw the line between what should be included in the decision problem and what should not be excluded. That is simply not an adequate decision-theoretical framework for policy or risk analysis, we think, since it does not help the decision-maker.^6^ Instead, problems have to be evaluated on a case-by-case basis, potentially making decisions more difficult and costly (Lundgren & Stefánsson, 2020; cf. Mumpower, 1986).^7^

## Normalcy, Probability, and Cost-effectiveness

In the previous section we discussed some problems for NDEUT related to arguments against the probabilistic *de minimis* principle. In this section, we will argue that NDEUT does worse in some cases than a probability-based *de minimis* principle because the probability of some terrible outcome need not be *de minimis* according to the probability-based *de minimis* principle even when it is sufficiently abnormal according to the normic *de minimis* principle. To illustrate this, let us diverge from the example used in the previous section and suppose we have a situation in which getting cancer is *abnormal*, but not *rare* (e.g., it may require an abnormal, but common, pre-condition). In such cases, NDEUT allows the decision-maker to ignore risks that we arguably should not ignore. Hence, applying NDEUT may result in treating an option with a high probability of someone dying as having only a *de minimis* risk.^8^

Note that our point is not that we cannot choose to *accept* the risk, but that it should not be permissible to ignore the risks just because they are abnormal. If the stakes are high, then even abnormal risks should be considered—especially if they involve high probabilities. Moreover, these types of examples do not seem to be rare. On the contrary, there seem to be many abnormal risks (some of which we discuss below) that must be given proper consideration.

There are two related problems here that we will discuss in turn. The first problem is that abnormality and cost-effectiveness come apart in various fairly common situations (as already illustrated by our modification of Lundgren and Stefánsson’s example). An illustrative example is medical decision-making. Many diseases are abnormal; indeed, most diseases are abnormal per definition given that they are divergences from normal bodily functions. However, their abnormality does not mean that we should be permitted to ignore them. Moreover, as pointed out by Lundgren and Stefánsson, even if a risk is unlikely, it can still be cost-efficient to avoid it or protect against it (see also Mumpower, 1986). This argument holds for abnormality as well. Consider, for example, diabetes. Diabetes is a metabolic disorder that results in increased blood sugar levels over extended periods. That is, it is—per definition—an *abnormal* metabolic behavior. Still, diabetes is very cost-efficient to treat.

Of course, one may argue that diabetes, despite being *physiologically abnormal*, is not *sufficiently abnormal* to be ignored. But we can take other examples. Two plausible candidates are Addison’s disease and Hypothyroidism. Both are abnormal disorders with fairly cheap treatments (Addison’s with cortisone and Hypothyroidism with thyroxine hormones) that, if untreated, can result in severe harm and even death. Although we could (again) question whether these disorders are sufficiently abnormal, that response-strategy would eventually call into question the whole notion of using normalcy to set the *de minimis* threshold. For any practically useful threshold that is set, we can presumably find a sufficiently abnormal disease that is still cost-effective to treat. (Of course, one could set a threshold such that it is *de minimis* only if it is maximally abnormal, but that makes the *de minimis* rule in the NDEUT almost always silent in practice, since maximally abnormal outcomes are so rare that they are hardly ever part of any *practical* decisional problem anyway.)

However, it might be possible to defend NDEUT by arguing that the exemplified diseases are *not* sufficiently abnormal *given the symptoms*. (Recall, from above, that Smith takes abnormality to be relative to an agent’s evidence.) In response to such an argument, we just have to consider a condition with no, or vague, symptoms; for example, early onset of certain forms of cancer or Lyme disease (the latter is caused by ticks and usually manifests itself with red markings around the bite, however, it can also manifest itself without such clear markings, in which case it can be difficult to identify simply because of its vague symptoms).

In response to the cancer example, it could be argued (at least for some forms of cancer) that cancer is not abnormal because it occurs as a matter of random fluctuation. So, in that sense, it is like losing or winning a lottery, neither of which is abnormal (i.e., neither of which calls for a special explanation). This goes to the very question of how we ought to understand the notion of abnormality. The way we read Smith’s bus example indicates that any delay of the bus is abnormal because it warrants a special explanation, which would then include random fluctuations in traffic patterns. Hence, random fluctuations could still be abnormal.^9^

Still, one may argue that a disease is not sufficiently abnormal given certain types of previous events, such as a lifetime of smoking or getting a tick bite. Being bitten by a tick might work like entering a Lyme-lottery (similarly, smoking might be like entering a cancer lottery). While that would avoid the above criticism of the normic *de minimis* principle, it would also suggest that normic risk does not track (decision relevant) risk appropriately since all outcomes in lotteries are equally normal. By contrast, getting bitten by a tick, say, causes risks (from discomfort to TBE and Lyme disease) that vary both in terms of probability and severity. Moreover, that does not resolve all of the outstanding problems, since there will be at least some diseases that cannot be considered to function like lotteries (i.e., that will be abnormal in the sense of demanding an explanation).

The second problem is that abnormality can also come apart from probability and disutility in various fairly common situations. That is, the normic conception of risk does not seem to be able to capture the notion of *de minimis*, understood as that which is sufficiently insignificant to be ignored. An illustrative set of examples are natural risks (such as accidents or pandemics), all of which seem to be abnormal per definition (of course, COVID-19 illustrates how a risk that was abnormal when it happened has since then become normal, which again illustrates the problem that normic risk does not appropriately track the decision relevant notion of risk). Indeed, for some of these examples, it seems that increased abnormality tracks increase disutility rather than the other way around. One illustrative example is that of radon.^10^ Radon is a radioactive gas, which exists naturally in varied amounts in different geographical locations. Radon can also exist in building materials. In particular, concrete made from material from geographic locations with high amounts of uranium can result in buildings with an increased level of radon. The normic conception of risk *prima facie* seems to correctly track the disutility and risks involved in some cases—for example, when radon is normal (e.g., naturally occurring) it is correctly identified as a risk that is not *de minimis*. However, even in geographical locations where radon is normal the occurrence of extreme levels of radon would be abnormal (even more so if there is an extreme exposure of radon in a geographical location where radon is not normally present). The problem is that extremely high levels of radon are more severe than more normal levels, so the normic risk conception does not track disutility in a way that is proper for a decision-making framework. Contrarily, this example illustrates that in some cases, the more severe the risk is, the more abnormal it would be. The problem, then, would be that however the threshold is set, NDEUT would consider some of the most extreme risks as *de minimis* simply in virtue of being abnormal. But clearly, the most extreme risks should not be treated as *de minimis*.

## Conclusions

In conclusion, we have shown that the normic conception of the *de minimis* principle and, more specifically, NDEUT leads to problematic violations of statewise dominance. Moreover, we have argued that NDEUT is in some sense worse than relying on the traditional probability-based *de minimis* principle because there will be severe risks and cost-efficient solutions that only the former will permit the decision-maker to ignore. Indeed, we have shown how NDEUT would permit the decision-maker to ignore the most severe risks in some situations.

## Data Availability

The manuscript has no associated data.

## Footnotes

1 Recently, Aven and Seif (2021) have argued that the *de minimis* principle should be complemented by an epistemic condition (according to which the knowledge that a risk falls beyond the threshold is “strong”). See Stefánsson and Lundgren (2022) for a response.

2 The online publication of Smith (2022) is without pagination.

3 This is similar to a discussion in Lundgren and Stefánsson (2020, p. 912) about whether to make a decision on a local or global level. Lundgren and Stefánsson show how this creates a dilemma for the traditional (i.e., probabilistic) understanding of *de minimis*.

4 The normic *de minimis* account has other virtues, Smith points out, such as solving what he calls the ‘lottery problem’ of the probabilistic *de minimis* account.

5 In some cases, a concern for *fairness* may justify violating statewise dominance (see, e.g. Stefánsson, 2015). But it should be evident from the above example that the *de minimis* principle sanctions violations of statewise dominance even in circumstances where fairness is not an issue. In other cases, a concern for respecting people’s different attitudes to risk may justify violating statewise dominance (see Bradley, 2022), and sometimes one may rationally violate statewise dominance when attitudes to risk change (see Stefánsson & Bradley, 2023). But just as we can construct examples in which fairness is not an issue, we can construct examples where risk attitudes are homogenous and constant. More generally, a *de minimis* threshold sanctions violating statewise dominance even in cases where we have no good justification for doing so.

6 Of course, using probabilistic measurements to complement NDEUT in determining what is *de minimis* would run into the same problems, as pointed out by Lundgren and Stefánsson (2020).

7 In fairness to Smith, we should note that his usage of the *de minimis* principle is not based on cost-effectiveness but his belief that adding it to NDEUT will resolve some problems for ordinary (normative) expected utility-based decision frameworks.

8 As Smith notes (2022, Sect. 5) risks with high probabilities can also generate problems for the probabilistic *de minimis* principle, since such risks can often be divided into sub-risks, each of which falls below the probabilistic *de minimis* threshold. (For a similar observation, see Lundgren & Stefánsson, 2020.)

9 Perhaps this goes to show that the notion of understanding abnormality *as events or outcomes that call for an explanation* might not be rich enough to capture the notion of normic risk. However, that is a question that goes beyond the scope of this paper. (Thanks to an anonymous reviewer for pushing us on these issues.)

10 This illustrates a potential similarity between, on the one hand, the normic *de minimis* principle in NDEUT, and, on the other hand, the idea that we should determine a probability-based *de minimis* threshold based on the level of *natural* risks (see, e.g., Weinberg, 1985). As Peterson (2002, p. 52) points out, radon cannot be considered negligible simply in virtue of being natural.
